# Novel mutations in *ADSL* for Adenylosuccinate Lyase Deficiency identified by the combination of Trio-WES and constantly updated guidelines

**DOI:** 10.1038/s41598-017-01637-z

**Published:** 2017-05-09

**Authors:** Xiao Mao, Kai Li, Beisha Tang, Yang Luo, Dongxue Ding, Yuwen Zhao, Chunrong Wang, Xiaoting Zhou, Zhenhua Liu, Yuan Zhang, Puzhi Wang, Qian Xu, Qiying Sun, Kun Xia, Xinxiang Yan, Hong Jiang, Shen Lu, Jifeng Guo

**Affiliations:** 1 Department of Neurology, Xiangya Hospital, Central South University, Changsha, Hunan P.R. China; 2 State Key Laboratory of Medical Genetics, Central South University, Changsha, Hunan P.R. China; 3 National Clinical Research Center for Geriatric Medicine, Changsha, Hunan P.R. China; 4 Key Laboratory of Hunan Province in Neurodegenerative Disorders, Central South University, Changsha, Hunan P.R. China; 5 Parkinson’s Disease Center of Beijing Institute for Brain Disorders, Beijing, P.R. China; 6 Collaborative Innovation Center for Brain Science, Shanghai, P.R. China; 7 Collaborative Innovation Center for Genetics and Development, Shanghai, P.R. China

## Abstract

Whole-exome sequencing (WES), one of the next-generation sequencing (NGS), has become a powerful tool to identify exonic variants. Investigating causality of the sequence variants in human disease becomes an important part in NGS for the research and clinical applications. Recently, important guidelines on them have been published and will keep on updating. In our study, two Chinese families, with the clinical diagnosis of “Epilepsy”, which presented with seizures, psychomotor retardation, hypotonia and etc. features, were sequenced by Trio-WES (including the proband and the unaffected parents), and a standard interpretation of the identified variants was performed referring to the recently updated guidelines. Finally, we identified three novel mutations (c.71 C > T, p.P24L; c.1387-1389delGAG, p.E463-; c.134 G > A, p.W45*; NM_000026) in *ADSL* in the two Chinese families, and confirmed them as the causal variants to the disease-Adenylosuccinate Lyase Deficiency. Previous reported specific therapy was also introduced to the patients after our refined molecular diagnosis, however, the effect was very limited success. In summary, our study demonstrated the power and advantages of WES in exploring the etiology of human disease. Using the constantly updated guidelines to conduct the WES study and to interpret the sequence variants are a necessary strategy to make the molecular diagnosis and to guide the individualized treatment of human disease.

## Introduction

As the rapid development and the huge decreased cost of next-generation sequencing (NGS) technologies, NGS has been used in an increasing number of studies for the research and clinical purpose of human disease^1, 2^. Exonic regions, account for about 1% of the genome, could cause approximate 85% of inherited disease^3^. Therefore, whole-exome sequencing (WES), which could well explore the exonic regions, has rapidly become a component of the clinical practice and is being applied to a wide range of clinical presentations that require a broad search for causal variants across the spectrum of genetically heterogeneous Mendelian disorders^4, 5^. However, when using the WES, it could offer about 25,000 variants in exome level per individual^6^. How to appropriately interpret the identified sequence variants and pinpoint the causal variants of the diseases are important parts in WES applications. Recently, The American College of Medical Genetics and Genomics (ACMG) has developed guidelines for the standard interpretation of sequence variants that cause Mendelian disorders^7, 8^. *Nature* ^9^ and the Clinical Sequencing Exploratory Research (CSER) consortium^10^ have also paid much attention to the standard interpretation of sequence variants as well.

Seizures and neurodevelopmental delay are common symptoms shared by a broad spectrum of diseases, and these genetically and clinically heterogeneous diseases are difficult to make a precise diagnosis with symptoms and signs alone in the clinical practice. In our study, we used Trio-WES (including the proband and the unaffected parents) and the related guidelines (Table 1) to explore them in Chinese population, and finally identified three novel mutations in *ADSL* gene in two Chinese families, and refined the diagnosis from “Epilepsy” to “Adenylosuccinate Lyase Deficiency”. This is the first report of *ADSL* mutations in Chinese population, which expanded the mutations spectrum of this gene. Our data also demonstrated that Trio-WES, combined with the objective interpretation of sequencing variants, is a preferable strategy to explore the genetics of human disease.

**Table 1:** The important guidelines for WES applications in our clinical applications.

No.	Title	Main content	Main applications	Main diseases	Ref.
1	Guidelines for investigating causality of sequence variants in human disease	Focus on investigating causality of sequence variants in human disease	Research and clinical applications	Rare and common diseases	9
2	Standards and guidelines for the interpretation of sequence variants: a joint consensus recommendation of the American College of Medical Genetics and Genomics and the Association for Molecular Pathology	Mainly focus on the interpretation of sequence variants in ever-known causative genes	Clinical applications	Mendelian disorders	7
3	Performance of ACMG-AMP Variant-Interpretation Guidelines among Nine Laboratories in the Clinical Sequencing Exploratory Research Consortium	Assess the performance of ACMG/AMP Variant-Interpretation Guidelines with detailed data	Clinical applications	Mendelian disorders	10
4	Consideration of Cosegregation in the Pathogenicity Classification of Genomic Variants	Improve on the detailed criteria on determining the “cosegregation” of ACMG	Clinical applications	Mendelian disorders	11
5	Recommendations for reporting of secondary findings in clinical exome and genome sequencing, 2016 update (ACMG SF v2.0): a policy statement of the American College of Medical Genetics and Genomics	Focus on the secondary findings in clinical exome and genome sequencing	Clinical applications	Mendelian disorders	8
6	Points to Consider: Ethical, Legal, and Psychosocial Implications of Genetic Testing in Children and Adolescents	Focus on the statement on genetic testing in children and adolescents	Research and clinical applications	Diseases in children and adolescents	22

## Results

### Clinical features

All of the four patients in the two families were partus matures with normal birth-weight. Then they began to present with neuropsychological clinical features characterized by severe psychomotor retardation, early onset of seizures, autism and etc.

**Figure 1:**
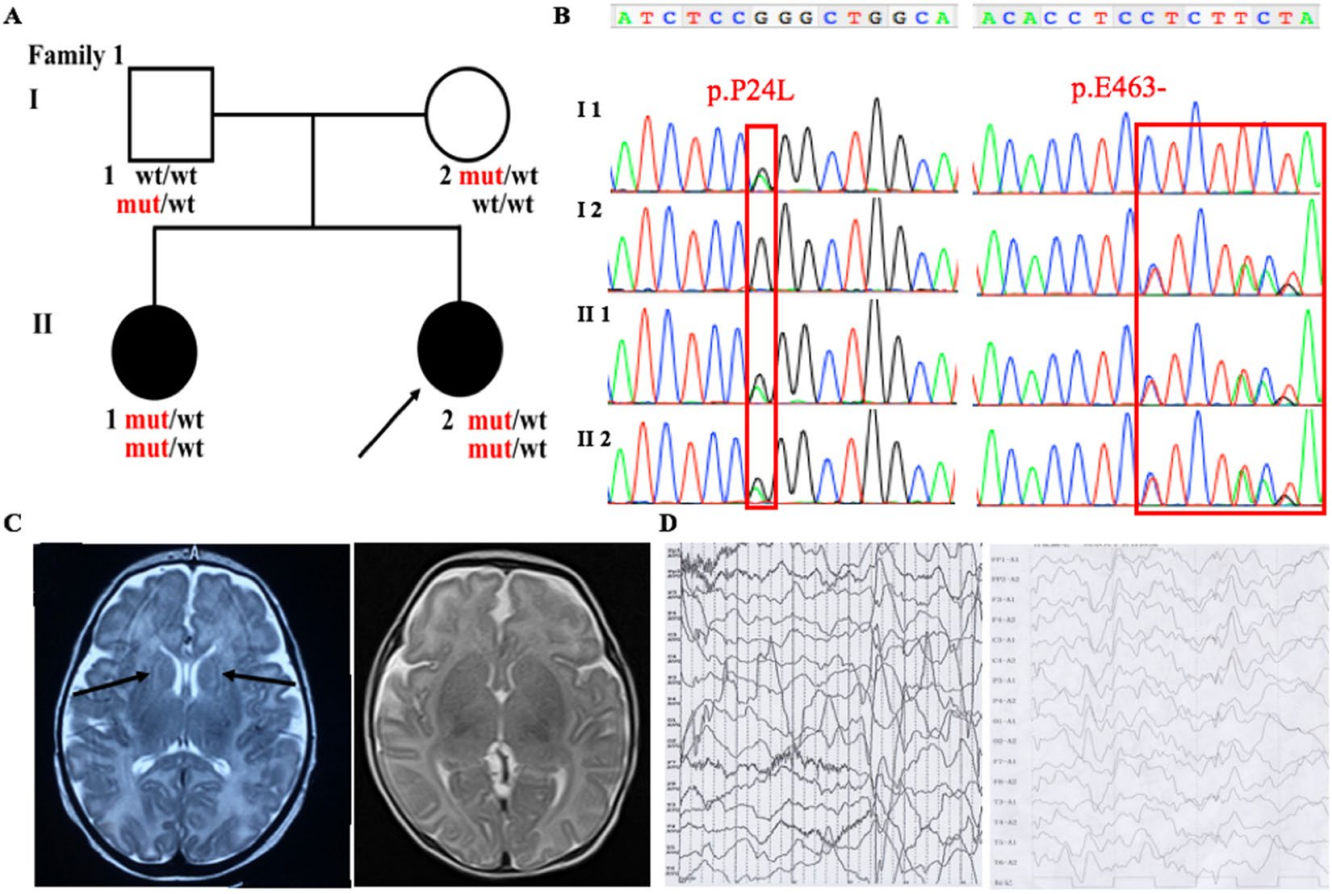
Clinical features of the patients and family 1 with the c.71 C > T, p.P24L; c.1387-1389delGAG, p.E463- in *ADSL*. (**A**) Pedigree structure of the studied family 1. In the family 1, WES was performed in I:1, I:2, II:2. Also, the compound heterozygous mutations were presented in the pedigree. (**B**) The PCR products were sequenced with the reverse primers (*ADSL* c.71 C > T, p.P24L; c.1387-1389delGAG, p.E463-). (**C**) The brain MRI shows the abnormal myelination of white matter in the proband (1-month old) (Left), and normal signal of the II 1 (Right) with T2-weighted. (**D**) EEG of the patients show the abnormal multifocal spike and ware wave in the proband (Left) and II 2 (Right).

In family 1 (Fig. 1A), the two patients have the similar features. The proband (II 2) is 0.5-year old now, while her sister (II 1) 2.5-year old. They firstly presented generalized seizures within 1-week old, which manifested with abnormal multifocal spike and ware wave in EEG (Fig. 1D). The average frequency of the seizures (including myoclonus) were 1–3 times per day before the treatment, however, it could reach to 8 times in some days. Valproate sodium and levetiracetam were adopted to them to control the seizures. Both II 1 and II 2 have marked psychomotor retardation, hypotonia and lack of eye-to-eye contact. Brain MRI examination showed obvious myelination in the white matter of the proband (II 2, 1-month old). No abnormal signals were observed in the brain MRI of the patient II 1 when she was 1-month old (Fig. 1C).

**Figure 2:**
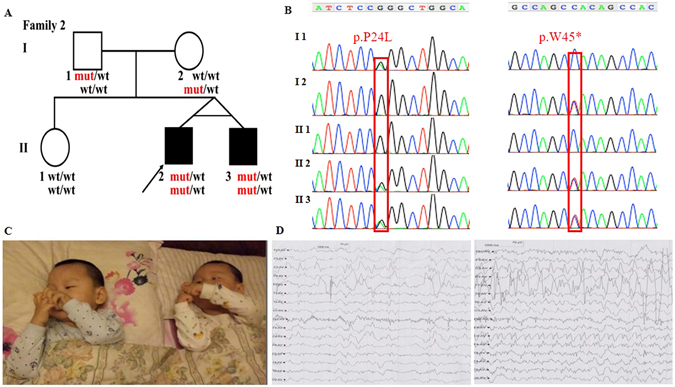
Clinical features of the patients and family 2 with the c.71 C > T, p.P24L; c.134 G > A, p.W45* in *ADSL*. (**A**) Pedigree structure of the studied family 2. In the family 2, WES was performed in I:1, I:2, II:2. Also, the compound heterozygous mutations were presented in the pedigree. (**B**) The PCR products were sequenced with the reverse primers (*ADSL* c.71 C > T, p.P24L; c.134 G > A, p.W45*). (**C**) Both of the patients have obvious hypotonia (could not sit by himself), autistic features (lack of eye-to-eye contact, repetitive movements, impaired verbal communication, etc.), etc. (3-year old). (**D**) EEG of the patients show the abnormal multifocal spike and ware wave in the proband (Left) and II 3 (Right).

The two patients in family 2 (Fig. 2A) are monozygotic twins who are 3-year old now. Similar to the family 1, they firstly presented generalized seizures within 1-week old, manifesting with abnormal multifocal spike and ware wave in EEG (Fig. 2D). The frequency of the seizures (including myoclonus) were 2–5 times per day before the treatment. Interestingly, there were some difference in phenotypes between the twins. For example, the clinical manifestation of II 3 was more severe than his elder brother and the two patients in family 1, such as showing recurrent temper tantrums, crying and poor sleep. Both II 2 and II 3 had marked psychomotor retardation, hypotonia and autistic features, etc. (Fig. 2C), which is similar to family 1. Although with different symptoms, they had same abnormal changes on brain MRI (2-month old), which presented with enlarged extracerebral space (MRI images were lost, with the scan reports left), indicating the atrophy of the cerebral cortex.

### Genetic findings

All of the four patients have a normal G-banding and karyotype. Using Trio-WES, more than 97% of the target regions were covered by at least 10 times for all the six individuals. After sequencing data analysis and variants filtering, we got 12 candidate genes with homozygous variants (*C1orf86, RNF19B, CYP2C18, BEST1, SLAIN1, IGHJ6, KCNC3, LILRA3, P2RY1, CANX, LCA10*), 5 candidate genes with compound heterozygous variants (*ADSL, RHOBTB1,TTN, HELZ2, SYNE1*), and 6 candidate genes with *de novo* variants (*FBLIM1, VPS33A, SMURF2, HIF3A, SCD5, HDAC9*) in family 1 (Supplementary Table S1); 7 candidate genes with homozygous variants (*AL359195.1, AGAP3, EGFL6, ZNF182, PFKFB1, ARHGAP36, MAMLD1*), 11 candidate genes with compound heterozygous variants (*ADSL, LPHN2, ABLIM1, DCHS1, A2ML1, PKD1L2, DCC, SIGLEC12, SLC3A1, ZFHX4, DMRT1*), and 8 candidate genes with *de novo* variants (*FOXO6, SKA3, ZFPM1, HES6, PIGU, CLCN2, TRBV5-4, CACNA1B*) in family 2 (Supplementary Table S2). Sanger sequencing was conducted with priority to the compound heterozygous variants (c.71 C > T, p.P24L and c.1387-1389delGAG, p.E463- in family 1; c.71 C > T, p.P24L and c.134 G > A, p.W45* in family 2;NM_000026) in *ADSL* (the only gene associated with seizures and neurodevelopmental delay among the candidate genes list above) for excluding the false positive of WES. The results of the Sanger sequencing are showed in Figs 1B and 2B. The novel variants in *ADSL* co-segregated well in the families (Figs 1A and 2A) and were not found in more than 60,000 people (Table 2). They are predicted as “damaging” by PolyPhen, SIFT, CADD or Mutation Taster (Table 2). According to ACMG guideline^7, 11^, our identified novel compound heterozygous variants are categorized to be “pathogenic variant” due to belonging to PVS1 (pathogenic very strong 1: null variant in a gene where loss of function is a known mechanism of disease), PS4 (pathogenic strong 4: the prevalence of the variant in affected individuals is significantly increased compared with the prevalence in controls), PM2-4 (pathogenic moderate 2–4: absent from controls, or at extremely low frequency if recessive, in Exome Sequencing Project, 1000 Genomes Project, or Exome Aggregation Consortium; for recessive disorders, detected in *trans* with a pathogenic variant; protein length changes as a result of in-frame deletions/insertions in a nonrepeat region or stop-loss variants), and PP1-3 (pathogenic supporting 1-3: cosegregation with disease in multiple affected family members in a gene definitively known to cause the disease; missense variant in a gene that has a low rate of benign missense variation and in which missense variants are a common mechanism of disease; multiple lines of computational evidence support a deleterious effect on the gene or gene product), which indicates that they are the etiology of the disease.

**Table 2:** The prediction of the identified variants in *ADSL*.

Gene name	Position	Transcript	Substitution	ExAC	1000 G	dbSNP ID	ESP	SIFT score	Polyphen score	Grantham score	GERP score	CADD score	Mutation Taster
*ADSL*	Chr 22: 40742633	NM_000026.2	c.71 C > T/p.P24L	novel	novel	novel	novel	0.067	0.001	98	0.153	14.87	Disease causing
*ADSL*	Chr 22: 40762457	NM_000026.2	c.1387-1389delGAG/p.E463-	novel	novel	novel	novel	NA	NA	NA	5.470	NA	Disease causing
*ADSL*	Chr 22: 40742696	NM_000026.2	c.134 G > A/p.W45*	novel	novel	novel	novel	NA	NA	NA	3.240	29.20	Disease causing

Abbreviations: ExAC, Exome Aggregation Consortium; 1000 G, 1000 genomes; ESP, Exome Sequencing Project; NA, not available.

### Secondary findings

As to the potential interest of the patients and medical actionability, informed consent of analyses of secondary findings (SFs) were got from the parents. The new updated SFs minimum list include 59 medically actionable genes recommended by the ACMG^8^. None of the known or expected pathogenic variants in theses genes were found in our patients.

### Specific therapy

After the molecular diagnosis of the patients, we refined the diagnosis from “Epilepsy” to “Adenylosuccinate Lyase Deficiency”. To date, there has no enzyme replacement therapy for Adenylosuccinate Lyase Deficiency, however, ketogenic diet^12^ and D-ribose^13^ were reported to be effective to some patients. In family 2, both II 2 and II 3 were given a ketogenic diet (a high-fat, adequate-protein, low-carbohydrate diet) for 14 months (beginning from 3-month old to 1.5-year old). During the ketogenic diet therapy, the seizures were eliminated without taking other antiepileptic drugs. However, the ketogenic diet therapy was finally given up and and replaced by sodium valproate due to the reoccurrence of the seizures. Patients in Family 1 did not adopt ketogenic diet therapy, but took levetiracetam and sodium valproate to control seizures. While taking the antiepileptic drugs, D-ribose administration was also introduced to both family 1 and 2, recently. D-ribose was administrated orally (four times daily) to the patients with an initial dose of 1 mmol/kg/day at beginning, then increased progressively up to 10 mmol/kg/day^13^. Interestingly, II 2 in family 2, who has the slightest features among the four patients, has an improvement in motor nimbleness and wish of playing and expressing himself after 1-month therapy. Now he has been treated with D-ribose for 3 months. However, II 3 in family 2 has no improvements after 1-month D-ribose administration, and gave it up. Both two patients in Family 2 gave up the D-ribose administration after 1-week treatment because of the increased frequency of seizures.

## Discussion

**Figure 3:**
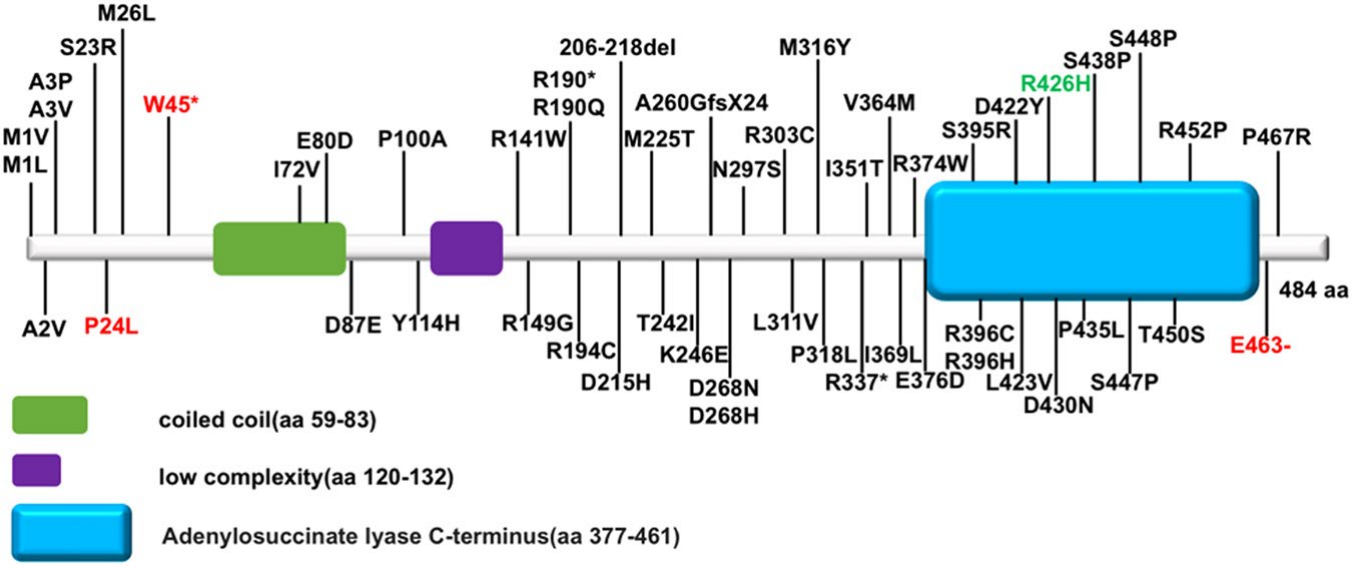
Mutations in ADSL protein. Note: aa, amino acid. Mutations in red are identified in our patients, mutation in green is the hot spot in European population. c.-49T > C, IVS5 -37 C > T and IVS12 + 1 G > C are not included in the figure. Schematic diagram was referred to SMART database (http://smart.embl-heidelberg.de). Mutations are referred to ADSL database (http://www1.lf1.cuni.cz/udmp/adsl/) and HGMD (http://www.hgmd.cf.ac.uk/ac/index.php).

Adenylosuccinate lyase deficiency (OMIM #103050) is an autosomal recessive defect of purine metabolism. Since the first mutation in *ADSL* reported in 1992^14^, several mutations have been reported in *ADSL* in European^15, 16^, however, there is few reports in Asian. In 2010, Chen *et al*. firstly reported two novel *ADSL* mutations in a Malaysian patient^17^. Here, we firstly reported three novel *ADSL* mutations in Chinese, which will expand the spectrum of mutations in *ADSL* in Asian. So far, there has been more than 50 mutations in *ADSL* been reported associating with Adenylosuccinate Lyase Deficiency (Fig. 3). Among these mutations, R426H was the most frequent one, indicating it is a hot spot in European^18^. The ever-known mutations locate in different parts/domains of the protein, and there is no minor allele frequency of missense/loss of function variants in *ADSL* larger than 0.005 in the Exome Aggregation Consortium (http://exac.broadinstitute.org), which may indicate the high conservation of *ADSL* sequence and its mutations predisposition to human disease.

Patients with Adenylosuccinate lyase deficiency were reported with significant clinical heterogeneity. In our patients, the clinical heterogeneity was also presented in clinical manifestations and brain MRI. For clinical manifestation, the symptoms of II 3 in family 2 was more severe (such as having recurrent temper tantrums, crying and poor sleep) than other patients, even than his twin-brother. Also, the patients’ brain MRI images showed different changes, without presenting corpus callosum missing, cerebellar vermis missing^19, 20^ or lissencephaly^21^, indicating the heterogeneity in brain MRI.

In the genetic test, we referred to the updated guidelines to ensure the accuracy of our study (Table 1). Our purpose of the test was according to American Society of Human Genetics recent suggestions for genetic testing in children and adolescents^22, 23^. Also, the attention should be paid when using WES, as nonstandard applications and interpretation of WES will cause serious effects on the doctor’s decision and patient’s therapy, etc.^24^. The process of our analysis and the interpretation of the sequencing variants are consisted to the guidelines published by MacArthur *et al*.^9^ and ACMG^7, 8^. Quality controls were conducted in the whole process, including DNA library creation, raw sequence data processing and base calling as described in previous paper^25^. After the standard quality controls of the WES, candidate genes associated with seizures and neurodevelopmental delay were preferentially considered to be the causative genes. As it is suggested by MacArthur *et al*.^9^ that “Investigators should begin by examining sequence variation in genes known to be associated with that phenotype, and assessing sequence coverage of the coding sequences and splice junctions for these genes before exploring the possibility of new candidate genes in the affected individuals”, Sanger sequencing was conducted with priority to *ADSL*. In the light of ACMG guidelines, we clinicians, laboratory scientists, and bioinformaticians, with collaborative interactions, objectively determined the identified novel variants in *ADSL* belong to “Pathogenic”, as they meet the criteria of PVS1, PS4, PM2-4 or PP1-3 in ACMG^7, 10, 11^, and then determined them to the causal mutations of the two families. The whole process of WES and the sequence variants interpretation in our study were standard.

Recently, secondary findings or incidental findings, which include genetic variants found incidentally or accidentally, have been paid much attention by the doctors, geneticist, and researchers^8, 26^. After obtaining the written informed consent from all the families, we also explored the secondary findings for the patients. Luckily, both the two probands have no identified mutations in 59 medically actionable genes suggested by ACMG^8^, indicating their low predisposition to the related disorders.

We also tried to conduct individual treatment after we refined the diagnosis from “Epilepsy” to “Adenylosuccinate Lyase Deficiency”. However, due to the limited research on Adenylosuccinate Lyase Deficiency therapy, no exciting effects were observed on all of our patients and the effect of D-ribose administration to Adenylosuccinate Lyase Deficiency was still ambiguous. This also indicates the eagerness to accelerate the evidence-based practice of genomic medicine and precision medicine^27^.

In conclusion, using Trio-WES, we identified three novel mutations in *ADSL* gene and using molecular diagnosis, we refined the diagnosis from “Epilepsy” to “Adenylosuccinate Lyase Deficiency”. This is a paradigm to do molecular diagnosis of rare Mendelian disease. Trio-WES, combining with the guidelines for investigating causality of sequence variants in human disease, has become a preferable option to explore the pathogeny and to make clinical practice of rare Mendelian disorders.

## Methods and Materials

### Subjects

More than 200 Chinese families with neurodevelopmental delay were enrolled in our study (data unpublished). In this manuscript, we reported two nonconsanguineous Chinese families (Figs 1A and 2A), and other than the four patients, there are no history of psychiatric or neurologic disorders reported in the families. Genomic DNA was extracted from peripheral blood leukocytes by standard phenol-chloroform extraction methods from the probands, and their unaffected parents and siblings. This study was approved by the ethics committee of Xiangya Hospital affiliated to Central South University and written informed consent was obtained from all the parents. Informed consent to publish identifying information/images was obtained from all the parents. The methods in this study were performed in accordance with the approved guidelines.

### Whole exome sequencing and Sanger sequencing

All of the four patients were conducted chromosome G-banding and karyotype analysis. After that, we took a Trio-WES strategy to identify the causal variants of the disease. TruSeq Exome Enrichment kit (Illumina) was used for exome capture and Hiseq 3000 platform (Illumina), with 2 × 150 bp pair-end reads, was used for sequencing the genomic DNA of the proband and the parents in WuXi AppTec (Shanghai, China).

Illumina’s Pipeline (version 1.3.4) with default parameters were used for raw image analysis and base calling. Sequence data were aligned to the reference human genome (hg19 version) using Burrows-Wheeler Aligner (BWA)^28^, duplicate reads were removed using Picard tool. We also used a comprehensive bioinformatics pipeline to analyze the exome sequencing data, including preprocessing, variant calling, annotation, and filtering according to our previous study^25^, and standard quality controls of the sequencing data were conducted in the process as well.

Considering the characteristics of the pedigree, the rare recessive, compound heterozygous and *de novo* variations in the probands were considered to be candidate causal variations with priority to the disease. Three rare novel mutations (c.71 C > T, p.P24L; c.1387-1389delGAG, p.E463-; c.134 G > A, p.W45*; NM_000026) in *ADSL* gene identified by WES were confirmed by Sanger sequencing. Two pairs of the primers for them are “Forward Primer TGGTCAAAGAAGCGAACCAA, Reverse Primer CGCAGCTCCTCAGGACAG” (c.71 C > T, p.P24L; c.134 G > A, p.W45*) and “Forward Primer TGTATTTGCTTTCCTCTGGCA, Reverse Primer AGGAAAGCACCATGGGAAGA” (c.1387-1389delGAG, p.E463-).

## Electronic supplementary material

Supplementary Table S1

Supplementary Table S2
